# Minoxidil Intoxication: A Case of Refractory Shock With Prolonged Supportive Care

**DOI:** 10.7759/cureus.73019

**Published:** 2024-11-04

**Authors:** Simon Maigre, Virginie Meurant

**Affiliations:** 1 Emergency Department, Centre Hospitalier Universitaire Tivoli, La Louvière, BEL

**Keywords:** 5% topical minoxidil, acute toxicology, medical intensive care unit (micu), minoxidil intoxication, refractory shock

## Abstract

Minoxidil, commonly used as a topical agent for alopecia, is occasionally used for refractory hypertension due to its antihypertensive properties. We report a rare case of massive minoxidil intoxication leading to refractory vasoplegic shock in a 24-year-old male. The patient ingested 120 mL of 5% minoxidil solution, resulting in vasoplegic shock. Management included fluid resuscitation and norepinephrine support. The patient required norepinephrine for 180 hours to maintain mean arterial pressure, a duration unprecedented in the literature. This case underscores the potential for prolonged vasopressor dependency following massive minoxidil ingestion and highlights the necessity for intensive symptomatic management. This report adds to the limited literature on minoxidil intoxication and suggests that extensive supportive care can be prolonged.

## Introduction

Minoxidil is a commonly used topical agent for the treatment of alopecia. In rare cases, it is employed in refractory hypertension due to its antihypertensive properties. Massive intoxication with minoxidil can lead to refractory vasoplegic shock, necessitating hospitalization and prolonged treatment in intensive care. We report the case of a 24-year-old man who presented with vasoplegic shock secondary to the voluntary ingestion of minoxidil, requiring prolonged vasopressor support in the intensive care unit.

## Case presentation

A 24-year-old male was admitted to the Emergency Department of the Tivoli University Hospital in La Louvière on August 18, 2024. The patient intentionally ingested 120 mL of 5% minoxidil topical solution two hours prior to his admission, with the intent of self-harm. Upon arrival, the patient presented with hypotension at 80/50 mmHg, mean arterial pressure at 60 mmHg, and a heart rate of 115 beats per minute. There was no sign of right nor left heart failure. The capillary refill time was noted to be 4 seconds, without associated mottling. Oxygen saturation was 98% in room air, and the respiratory rate was 20 breaths per minute, with no clinical signs of respiratory distress. Pulmonary auscultation revealed no abnormalities. The patient was alert and did not exhibit any signs of encephalopathy.

Arterial blood gas analysis indicated a compensated metabolic acidosis with respiratory alkalosis. The pH was 7.38, PCO_2_ was 30 mmHg, PO_2_ was 95 mmHg, and lactate level was 4 mmol/L. Laboratory tests showed a troponin level of 0.03 ng/mL, rechecked at 3 hours as 0.04 ng/mL. There was no sign of renal or hepatic insufficiency, and electrolyte levels and coagulation parameters were within normal limits (Table 1).

**Table 1:** Blood test results

Test	Value	Unit
Complete blood count		
Red blood cell count	3.9	10^6^/µL
Hemoglobin	13.4	g/dL
Hematocrit	42.0	%
Mean corpuscular volume	88.0	fL
Mean corpuscular hemoglobin	29.0	pg
Mean corpuscular hemoglobin concentration	33.5	g/dL
White blood cell count	9700.0	10^3^/µL
Platelets	348.0	10^3^/µL
Leukocyte formula		
Neutrophils	62.0	%
Lymphocytes	28.0	%
Monocytes	6.0	%
Eosinophils	3.0	%
Basophils	1.0	%
Biochemistry		
Glucose	112.0	mg/dL
Creatinine	0.7	mg/dL
Urea	28.0	mg/dL
Uric acid	4.0	mg/dL
Total protein	7.2	g/dL
Albumin	4.8	g/dL
Liver enzymes		
Alanine aminotransferase	26.0	U/L
Aspartate aminotransferase	19.0	U/L
Alkaline phosphatase	64.0	U/L
Gamma-glutamyl transferase	27.0	U/L
Bilirubin total	0.7	mg/dL
Electrolytes		
Sodium	139.0	mmol/L
Potassium	3.8	mmol/L
Chloride	98.0	mmol/L
Calcium	9.4	mg/dL
Magnesium	1.8	mg/dL
Phosphate	3.4	mg/dL
Coagulation		
Prothrombin time	14.0	seconds
Activated partial thromboplastin time	28.0	seconds
International normalized ratio	0.92	ratio
Fibrinogen	210.0	mg/dL
Cardiac biomarkers		
Troponin I	0.03	ng/mL

**Figure 1:**
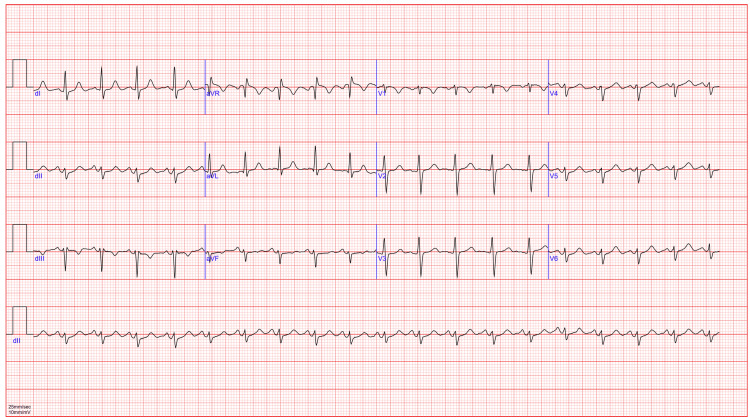
Electrocardiogram

The electrocardiogram revealed a regular sinus rhythm with a heart rate of 115 beats per minute, without conduction or repolarization disturbances (Figure 1).

Initial management consisted of catheter insertion with two peripheral intravenous lines and intravenous fluid resuscitation with 3,000 mL of 0.9% NaCl. Despite fluid resuscitation, hypotension persisted at 90/50 mmHg, tachycardia at 115 beats per minute, and hyperlactatemia at 3 mmol/L. The patient was transferred to the intensive care unit, where norepinephrine was initiated as vasopressor support. A cardiac ultrasound performed upon admission showed a hyperdynamic left ventricle with an ejection fraction estimated at 75%, low left ventricular filling pressures, an aortic velocity time integral (VTI) of 25 cm, and excellent right ventricular function. No valvular abnormality or pericardial effusion was noted. The inferior vena cava appeared collapsible.

**Figure 2:**
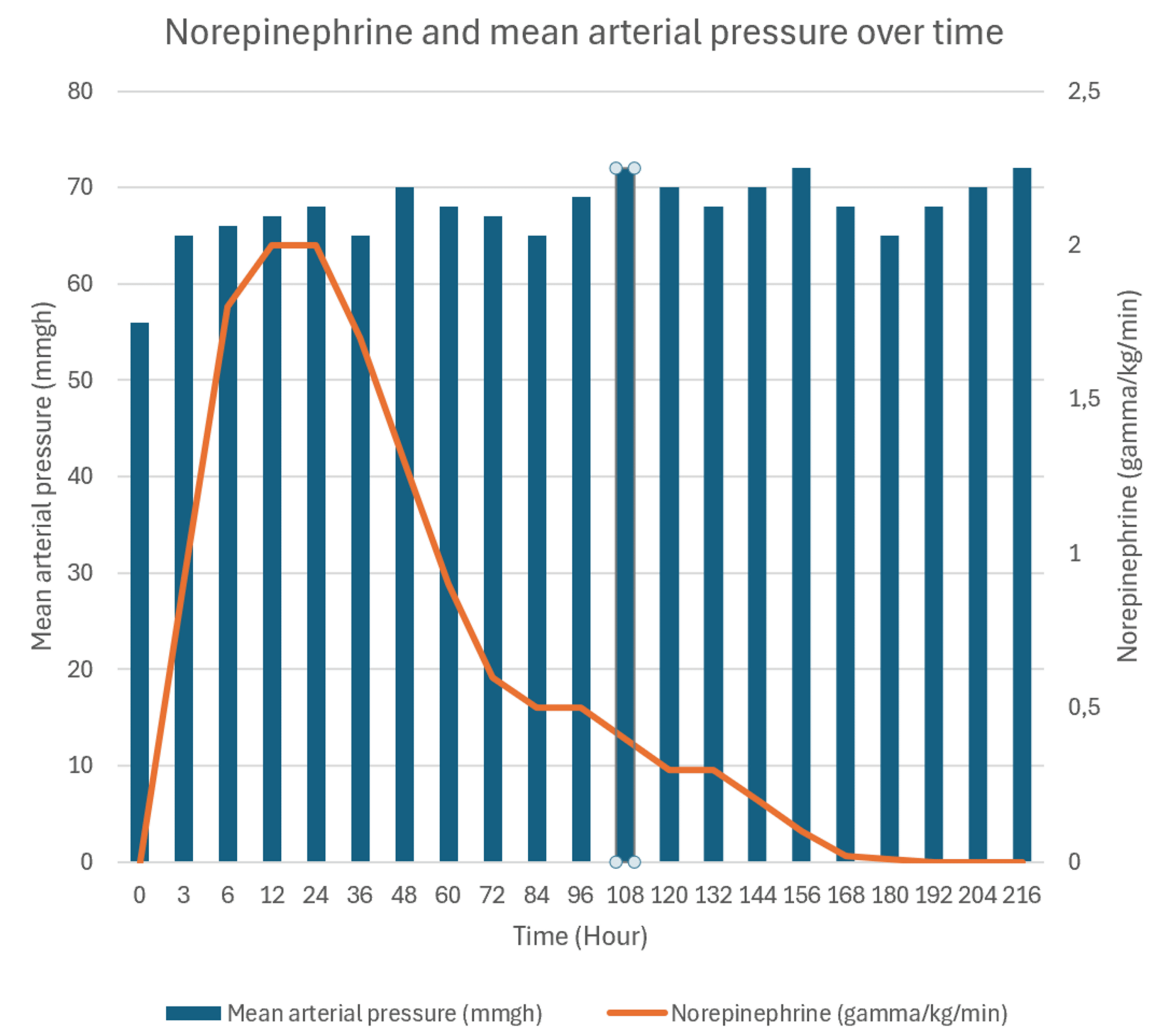
Norepinephrine and mean arterial pressure over time

The patient remained dependent on norepinephrine monotherapy at doses of up to 2 µg/kg/min for 180 hours to maintain a mean arterial pressure of 65 mmHg. Lactate levels normalized within hours of admission. The patient did not experience any further organ failure during his stay in the intensive care unit, including no renal insufficiency, hepatic insufficiency, or encephalopathy. The doses of norepinephrine and the mean arterial pressure over time during hospitalization are shown in Figure 2.

He was discharged from the unit after psychiatric evaluation on the ninth day of hospitalization.

## Discussion

Minoxidil has been known since the 1960s for its hypotensive properties [1]. Its efficacy in treating hair loss was an incidental discovery, leading to the development of a topical formulation. In Belgium, two commercial concentrations of minoxidil are available in 60 mL bottles: 2% and 5%.

Only a few cases of minoxidil intoxication have been reported in the literature. The uniqueness of our case lies in the massive dose ingested and the duration of vasopressor support significantly exceeding references found in existing literature [2-6]. Minoxidil is 95% absorbed by the gastrointestinal tract, with peak plasma levels reached within 1 hour. It undergoes hepatic metabolism via sulfotransferase to become its active form, minoxidil sulfate. Minoxidil sulfate activates ATP-modulated K+ channels, resulting in hyperpolarization and relaxation of smooth muscle. The maximal hypotensive effects may be delayed due to the lag in forming the active metabolite. Minoxidil has a plasma half-life of 3-4 hours, but its length of action can last 24 hours or occasionally longer, possibly due to its persistence in vascular smooth muscle [7].

Systemic dosages for antihypertensive treatment typically range from 10 to 40 mg per day [8]. In this case, the absorbed dose was 6,000 mg, representing 150 to 600 times the usual therapeutic range. The literature does not provide comparable dosages [2-6]. This massive dose likely explains the prolonged duration of norepinephrine support monotherapy for 180 hours. Based on the reviewed literature, such an extended duration of vasopressor support is unprecedented [2-6]. The hypothesis is that the redistribution of minoxidil within smooth muscle may account for the prolonged effect [7].

Our patient did not receive activated charcoal treatment upon initial management. Given the time elapsed between ingestion and admission, the rapid absorption of minoxidil, and the absence of an enterohepatic cycle for this molecule, this treatment was not administered, in line with recent recommendations [9].

Our case demonstrates that prolonged symptomatic management may be necessary in cases of massive minoxidil intoxication. Notably, there was no associated organ failure, and there was no need for a second-line vasopressor, which was considered during the hospitalization.

## Conclusions

Minoxidil intoxication, as a potent vasodilator and antihypertensive agent, can result in refractory vasoplegic shock, requiring intensive care admission. Treatment is symptomatic, focusing on volume optimization and tailored vasopressor support. This treatment may need to be prolonged, especially in cases of massive ingestion.

## References

[1] Farrell SE, Epstein SK (1999). Overdose of Rogaine Extra Strength for Men topical minoxidil preparation. J Toxicol Clin Toxicol.

[2] Dash S, Sashindran VK (2023). Minoxidil poisoning: a case of refractory shock with remarkable ECG changes. Indian J Crit Care Med.

[3] McCormick MA, Forman MH, Manoguerra AS (1989). Severe toxicity from ingestion of a topical minoxidil preparation. Am J Emerg Med.

[4] Shashikala TP, Singh R, Muthukrishnan J (2016). Refractory shock following ingestion of topical minoxidil solution. Med J Armed Forces India.

[5] Gheshlaghi F, Zoofaghari S, Dorooshi G (2018). Unstable angina: a rare presentation of minoxidil intoxication: a case report and literature review. J Res Pharm Pract.

[6] MacMillan AR, Warshawski FJ, Steinberg RA (1993). Minoxidil overdose. Chest.

[7] Brunton Brunton, Laurence L, Chabner B (2011). Goodman & Gilman's the pharmacological basis of therapeutics.

[8] Poff SW, Rose SR (1992). Minoxidil overdose with ECG changes: case report and review. J Emerg Med.

[9] Mégarbane B, Oberlin M, Alvarez JC (2020). Management of pharmaceutical and recreational drug poisoning. Ann Intensive Care.

